# Prevalence of Vitamin A Deficiency among Preschool Children in Ethiopia: A Systematic Review and Meta-Analysis

**DOI:** 10.1155/2020/8032894

**Published:** 2020-02-27

**Authors:** Zekariyas Sahile, Delelegn Yilma, Robel Tezera, Tadu Bezu, Werissaw Haileselassie, Benyam Seifu, Jemal Haidar Ali

**Affiliations:** ^1^Ambo University, College of Medicine and Health Science, Department of Public Health, P.O. Box 19 Ambo, Ethiopia; ^2^Addis Ababa University, College of Health Science, Department of Medical Radiological Technology, P.O. Box 11950 Addis Ababa, Ethiopia; ^3^Kotebe Metropolitan University, Menelik II Health Science and College of Medicine, Addis Ababa, Ethiopia; ^4^Addis Ababa University, College of Health Sciences, School of Public Health, P.O. Box 11950 Addis Ababa, Ethiopia; ^5^Ambo University, College of Medicine and Health Science, Department of Midwifery, P.O. Box 19 Ambo, Ethiopia; ^6^Addis Ababa University, College of Health Science, School of Public Health, P.O. Box 27285 1000 Addis Ababa, Ethiopia

## Abstract

**Background:**

Vitamin A deficiency is a major nutritional concern in lower-income countries. The aim of this systematic review and meta-analysis was to show the magnitude of vitamin A deficiency among preschoolers in Ethiopia.

**Objective:**

The present study was aimed at synthesizing qualitatively and quantitatively the existing literature on the prevalence of VAD in preschool children in Ethiopia.

**Methods:**

Studies were searched through the search engine of Google Scholar, Hinari, MEDLINE/PubMed, Cochrane Library, and Africa-Wide Information. Searching was made using the keywords/MeSH of vitamin A deficiency, xerophthalmia, night blindness, Bitot's spot, retinol, children, and Ethiopia. Data were analyzed and compared with the WHO threshold criteria to declare a public health problem. Heterogeneity among studies was assessed using a Cochran *Q* test and *I*^2^ statistics. A random-effects model with 95% confidence interval was used for prevalence estimations.

**Results:**

Of the 13 studies included in clinical analysis, 12 of them reported the prevalence of night blindness and/or Bitot's spot among preschool children in Ethiopia which was above WHO cutoff point for the public health problem 1% and 0.5%, respectively. The prevalence of night blindness significantly decreased from moderate public health problem 4.2% (95% CI: 2.8%-5.7%) in a period from 1990 to 2004 to mild public health problem 0.8% (95% CI: 0.6%-1.0%) in a period from 2005 to 2019. Furthermore, statistically insignificant reduction was observed in the prevalence of Bitot's spot in a period from 1990 to 2004, 2.2% (95% CI: 1.3%-3.2%) to 1.8% (95% CI: 1.2%-2.3%) in a period from 2005 to 2019. Among 8 studies on subclinical vitamin A deficiency, 7 of them indicated a severe public health problem (>20%). The prevalence of subclinical vitamin A deficiency decreased from 55.7% (95% CI: 39.8%-71.6%) in a period from 1990 to 2004 to 28.3% (95% CI: 9.8%-46.7%) in a period from 2005 to 2019, but not statistically significant.

**Conclusions:**

Despite the reduced proportion of night blindness and Bitot's spot, still both clinical and subclinical vitamin A deficiencies remain a public health problem in Ethiopia requiring strengthen intervention through the newly initiated health extension program.

## 1. Introduction

Vitamin A deficiency (VAD) is a major nutritional concern in poor societies, especially in lower-income countries. Its presence as a public health problem is assessed by measuring the prevalence of deficiency in a population, represented by specific biochemical and clinical indicators of status [1].

Two sets of indicators are commonly used to assess the magnitude of VAD in population surveys and include ocular and serum or plasma retinol assessment. Xerophthalmia ranges from milder stages of night blindness and Bitot's spots, to potentially blinding stages of corneal xerosis, ulceration, and necrosis. Although ocular assessment varies and advanced lesions involving the cornea are rare, early lesions that include night blindness and Bitot's spots are most commonly used for the assessment of the severity of the problem. Night blindness is obtainable by history and Bitot's spots observable by hand light examination of the conjunctiva surface [2]. The second approach is measuring serum retinol concentrations to assess subclinical vitamin A status in a population, with values below 0.70 *μ*mol/l indicating VAD [3].

In Africa, 2% of preschool-age children are affected by night blindness which is four times higher than the proportion in South East Asia (0.5%). Moreover, almost half of the children affected globally are found in Africa (1). VAD alone is responsible for almost 6% of child deaths under the age of 5 years in Africa [4]. According to World health Organization (WHO) estimates, the prevalence of night blindness in preschool children in Ethiopia from 1995 to 2005 was 4.9% and an estimated 658,000 preschoolers were diagnosed with VAD. The survey indicated VAD as a moderate public health problem in Ethiopia [1]. According to the 2014 global nutritional report, in Ethiopia, subclinical vitamin A deficiency in the same age groups of children was 46% for the period from 1996 to 1997 [5].

Vitamin A supplementation (VAS) was found effective in the reduction of morbidity and mortality [6, 7]. A systematic review using African studies showed that vitamin A supplementation in children 6–59 months of age is associated with 24% reduction in under-five child mortality rate and reduced incidence of diarrhea and measles [6]. VAS also reduced the severity and fatality from measles, diarrhea, and prevalence of night blindness and xerophthalmia [6, 7]. A study conducted in Tigray region of Ethiopia among preschool children found that vitamin A supplementation significantly reduced the prevalence of Bitot's spot (1%), fever (15.6%), diarrhea (12%), oedema (6%), measles (7.8%), and conjunctivitis (7.2%). Moreover, children retinol concentration was also improved [8]. Despite recent papers reporting that improved coverage of immunizations and better management of diarrhea and measles altered the effectiveness of VAS and that periodic VAS has minimal impact on subclinical VAD and under-five mortality rate [9, 10], in the 2017 systematic review, VAS was still associated with a significant 12% reduction of under-five mortality [7].

Vitamin A supplementation is a relatively short-term, low-cost, highly effective strategy for improving the vitamin A status of children from 6 to 59 months of age. VAS is being administered as per WHO guidelines [11]; for infants 6 to 11 months, 100,000 I.U. is being administered once, and for children 12 to 59 months, 200,000 I.U. is given every 4 to 6 months. Vitamin A should not be given in less than one-month intervals, and all vitamin A supplementation must be recorded on each child's growth monitoring or other cards to avoid duplicate dosing. Supplementation of vitamin A capsules takes place during mass supplementation campaigns (twice a year) and routine health service delivery including immunization sessions [12]. In Ethiopia, only 45% of children ages 6-59 months received vitamin A supplements in 2016 [13].

The effectiveness of interventions to reduce VAD was measured by assessing the prevalence of VAD. There is limited recent information on national prevalence of VAD in preschool children in Ethiopia. Since 1990, two national surveys of VAD were conducted in 2005 [14] and in 2015 [15] that reported 37.7% and 13.9% national prevalence of VAD, respectively. One summary of the literature review of vitamin A deficiency among children and women was documented from 1957 to 2005 [16] indicated that VAD has remained as a major public health problem in Ethiopia. The last review is more than 10 years old. Hence, further systematic review and meta-analysis is necessary to show the pattern of prevalence. Thus, showing the pattern of vitamin A deficiency has implication for considering different interventions to reduce the deficiency. Therefore, this systematic review and meta-analysis was conducted to generate evidence-based national usable information for policy makers and other stakeholders including health care providers to reduce the burden of vitamin A deficiency in the country. In general, this study was aimed at synthesizing qualitatively and quantitatively the existing literature on the prevalence of VAD in preschool children in Ethiopia.

## 2. Methods

### 2.1. Eligibility Criteria

Cross-sectional studies which reported the prevalence of VAD in preschool children in Ethiopia were considered eligible for this systematic review and meta-analysis. Other eligibility criteria were studies that measured vitamin A deficiency using subclinical and/or clinical assessment methods among preschoolers age 6 months to 6 years in the period from 1990 to 2019. Searching was made from the first of January to end of March 2019. Studies were excluded if they were not primary studies (such as review articles, conference abstract, and editorials). In addition, studies conducted among malnourished preschooler children and/or any other disease were used as exclusion criteria.

### 2.2. Information Source

A search was made using databases of Google Scholar, Hinari, MEDLINE/PubMed, Cochrane Library, Africa-Wide Information, African Index Medicus, and other sources like contacting experts and researchers to retrieve new articles and by manual searching to identify unpublished studies. This was carried out by hand search by going to universities and research institution libraries to see a card catalog of relevant studies.

### 2.3. Search Strategy

Searching was made using keywords/MeSH of “Vitamin A deficiency”, “xerophthalmia”, “night blindness”, “Bitot's Spot”, “retinol”, “children”, and Ethiopia.

### 2.4. Selection Process

Selections of the studies were conducted based on the Preferred Reporting Items for Systematic review and Meta-Analysis (PRISMA) guideline [17]. The studies were screened using eligibility criteria by two authors independently and crosschecked for the consistency. Moreover, discrepancies between authors were resolved through repeated evaluation, discussion, and consensus. This systematic review and meta-analysis was not registered with PROSPERO.

### 2.5. Data Extraction Process

An agreed abstraction format was developed to extract data from selected studies. The data extraction format has the following structure: author details (name and year of publication), study year, study setting, study design, sample size, study population, sampling procedures, data collection procedures, and findings. The data were extracted independently by two authors, and results were compared to check the consistency. In case of inconsistency, the articles were reviewed again and disagreements were resolved by verification and further discussion.

### 2.6. Outcome Variables

The outcome variables for this systematic review and meta-analysis were vitamin A deficiency through clinical examination (night blindness and Bitot's spot) and subclinical analysis (serum retinol concentration). Night blindness, a condition in which a child cannot see in dim light, is generally the earliest clinical manifestation of vitamin A deficiency. Indirect outcome (mothers/caregivers reported a child cannot see in dim light) was used in all clinical studies to assess night blindness and Bitot's spots—opaque whitish deposits on the scleral conjunctiva is the most characteristic sign of ocular problems related to vitamin A. Bitot's spot was assessed based on eye examination using an ophthalmoscope to examine children with collections of keratin in the conjunctiva with a small cheesy or foamy ocular lesion overlaying a patch of rough or xerotic conjunctiva. Serum retinol concentration < 0.70 *μ*mol/l indicates subclinical VAD and a serum retinol value of <0.35 *μ*mol/l indicates severe VAD.

### 2.7. Risk of Bias Assessment and Quality of Evidence Assessment

Risk of bias assessment of the studies was carried out by two authors independently using the Hoy 2012 tool with ten criteria: (1) representation of the population, (2) sampling frame, (3) methods of participants' selected, (4) nonresponse bias, (5) data collection directly from subjects, (6) acceptability of case definition, (7) reliability and validity of study tools, (8) mode of data collection, (9) length of prevalence period, and (10) appropriateness of numerator and denominator. Items 1 to 4 assess selection and nonresponse bias, items 5 to 9 assess measurement bias, and item 10 assesses bias related to analysis. Each item was assessed as either low or high risk of bias, and the overall risk of bias was defined based on the score of high-risk items to bias per study: low (≤2), moderate (3–4), and high (≥5) [18].

An evaluation of the degree of certainty of the evidence for each outcome studied was performed using the GRADE (Grading of Recommendations Assessment, Development and Evaluation) tool. In the GRADE approach, observational studies start from low-quality evidence but downgraded to very low based on the five factors: risk of bias, inconsistency, indirectness, imprecision, and publication bias. Evidence from observational studies can be upgraded provided no other limitations have been identified based on the five factors. The GRADE tool has three additional upgrading domains, evidence of dose-response association, all possible confounding taken in account, and large magnitude of effect. Assessments were made for the five main domains (risk of bias, consistency, directness, precision, and publication bias), as well as overall quality of evidence. We used study design as our starting point and downgraded by one step for each domain that was not met, based on the GRADE recommendation [19].

### 2.8. Statistical Analysis and Synthesis

Extracted data were entered and analyzed using Review Manager (RevMan) version 5.3 statistical software. The variance of vitamin A deficiency (VAD) prevalence for each article was calculated based on the binomial distribution formula by extracting the frequency of outcome and sample size [20]. Detail synthesis was made regarding the prevalence of VAD using both clinical (night blindness and Bitot's spot) and subclinical (serum retinol concentration < 0.70 *μ*mol/l) methods by different time periods and area of study. Results were compared with WHO cutoff point for the level of public health importance. According to the WHO, VAD is a public health problem in which 1% is for night blindness and 0.5% for Bitot's spot [21]. For subclinical VAD (serum retinol levels less than 0.7 *μ*mol/l), a range from 2% to 10% is mild, 10% to 20% is moderate, and more than 20% is severe public health problem [22].

Heterogeneity among studies was assessed using a Cochran *Q* test (*P* value < 0.10 is considered significant) and *I*^2^ statistics (at least 50% is considered significant) [23]. A random-effects model with 95% confidence interval (CI) was used for estimation because significant variations were shown between the study findings. The random-effects model is more conservative than the fixed-effects model and helps to account heterogeneity inherent in meta-analysis. Subgroup analysis was performed based on the quality of studies, sex, and survey period. Funnel plot analysis, Egger weighted regression, and Begg rank correlation tests were used to detect publication bias, and *P* value < 0.05 was considered indicative of statistically significant publication bias. The findings are depicted using forest plots and tables.

## 3. Results

### 3.1. Characteristics of the Studies

**Figure 1:**
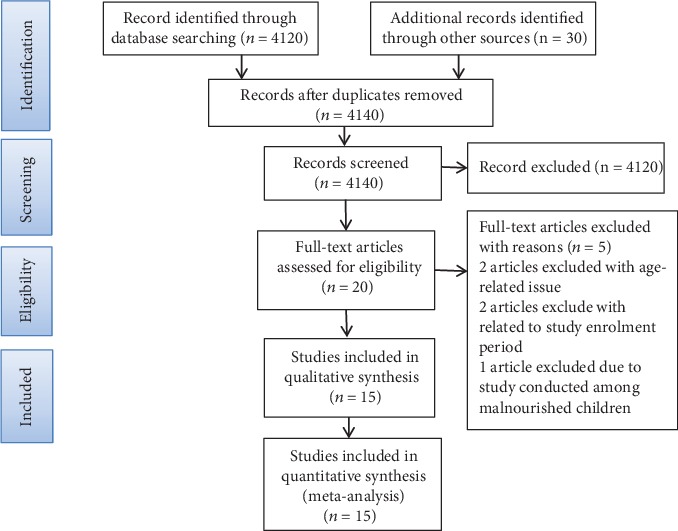
PRISMA flow chart diagram describing selection of studies for systematic review and meta-analysis on the prevalence of vitamin A deficiency among preschool children in Ethiopia 1990-2019.

A total of 4,150 published and unpublished studies were obtained through searching from different databases. Out of 4,150 studies, 10 studies were excluded because of duplication, and 4,120 studies were excluded after reading the title and abstract using inclusion and exclusion criteria. The remaining 20 full-text studies were assessed for eligibility. Finally, 15 studies were included in systematic review and meta-analysis (Figure 1).

All 15 studies included in this systematic review and meta-analysis were community-based cross-sectional studies. Seven studies used both clinical and subclinical (biochemical) assessment methods [8, 16, 24–28], and six of them used clinical assessment method only [29–34], while two studies were subclinical assessment method only [15, 35]. Thirteen studies indicated the prevalence of xerophthalmia through clinical examination. About 56,597 preschool children (age 6 to 72 months) were included in the clinical examination from thirteen studies, and 3,770 preschool children were included in the subclinical analysis of eight studies. The earliest and the latest studies conducted were in 1991 [29] and 2016, respectively [35] (Table 1).

### 3.2. Risk of Bias and Heterogeneity

The risk of bias assessment of individual studies was carried out using the Hoy 2012 tool with ten criteria which were listed in Methods [18]. Out of 15 included studies, eight studies (53.3%) had a low risk of bias [14, 15, 25, 26, 31, 33, 35], and seven studies (46.7%) had a moderate risk of bias [8, 24, 27–30, 34]. The main cause of study bias was selection bias. High risk of selection bias was observed in eight studies. Measurement bias was observed in three studies [28, 32, 34]. Bias related to analysis was also observed in three studies [8, 27, 33] (Supplementary file 1: [Supplementary-material supplementary-material-1]).

**Figure 2:**
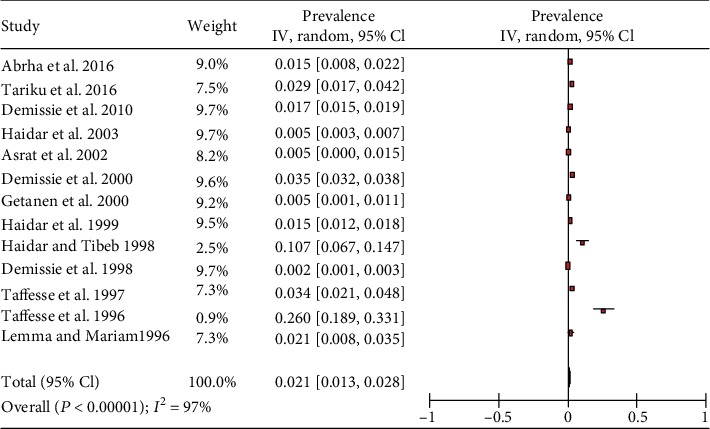
Forest plot of 13 studies on the prevalence of Bitot's spot among preschool children in Ethiopia, 2019.

**Figure 3:**
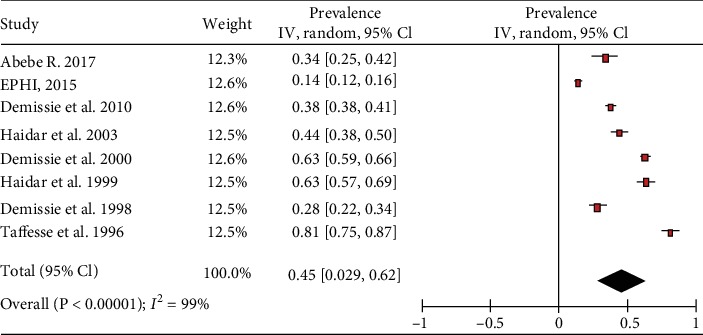
Forest plot of 8 studies on the prevalence of subclinical vitamin A deficiency among preschool children in Ethiopia, 2019.

**Figure 4:**
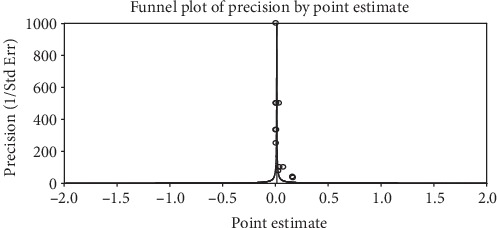
A funnel plot of studies conducted on the prevalence of night blindness in Ethiopia.

**Figure 5:**
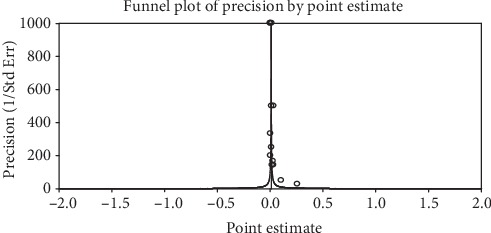
A funnel plot of studies conducted on the prevalence of Bitot's spot in Ethiopia.

**Figure 6:**
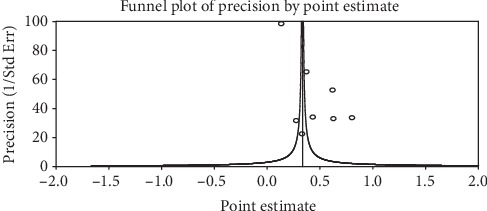
A funnel plot of studies done on the prevalence of subclinical vitamin A deficiency in Ethiopia.

The included studies exhibited high heterogeneity according to Cochrane *Q* test (*Q* test *P* = 0.00001, *I*^2^ test (97%)) in 13 studies that used clinical examination and Cochrane *Q* test (*Q* test *P* = 0.00001, *I*^2^ test (99%)) in 8 studies that used subclinical VAD assessment which is an indication to use the random-effects model (Figures 2 and 3). However, the Egger weighted regression statistics of studies conducted on the prevalence of night blindness (*P* = 0.052), Bitot's spot (*P* = 0.079), and subclinical vitamin A deficiency (*P* = 0.078) and Begg rank correlation statistics (*P* ≥ 0.05) indicated no evidence of publication bias. There was no sign of publication bias and asymmetry in the funnel plot (Figures 4–6). To reduce the heterogeneity, subgroup analysis was performed based on the quality of studies, survey year, and sex. In studies conducted from years 2005 to 2016 for clinical assessment, there was low heterogeneity (11%) for night blindness and moderate heterogeneity (48%) for Bitot's spots. Nonetheless, the heterogeneity in all subgroups was considerable (Table 2).

According to GRADE evidence tool assessment, the overall quality of evidence for night blindness was found to be very low due to risk of bias, inconsistency, and indirectness. The overall quality of evidence for Bitot's spot was very low due to risk of bias and inconsistency, whereas the quality of evidence for subclinical VAD was found to be very low due to inconsistency (supplementary file 2: [Supplementary-material supplementary-material-1]).

### 3.3. Prevalence of Clinical Vitamin A Deficiency (Xerophthalmia)

A total of 13 studies were included in this systematic review and meta-analysis to show the prevalence of xerophthalmia. A study conducted in Hararge region of Eastern Ethiopia showed that prevalence of night blindness among preschool-age children was 16.5% in 1991 [29]. Based on three studies conducted in 1993, the prevalence of night blindness ranged from 4.2% in Southwest Ethiopia [30] to the highest prevalence of 17% in Oromia region, central Ethiopia [24]. Three studies conducted in 1996 indicated that the prevalence of night blindness was 0.9% in the Tigray region [25], 1.2% in the Southern region [26], and 8.4% in the Harari region [27], whereas in studies conducted in 1997, it was 0.8% in Tigray region [8] and 3.2% in Oromia region [28]. The national prevalence of child night blindness was 0.8% in 2005 with high rate in the Harari region (1.1%) followed by the Amhara and Benishangul-Gumuz (both 1.0%) [14]. Likewise, about 1.2% of child night blindness was documented in the rural Tigray region in 2014 [32] and the lowest prevalence (0.6%) was reported in Amhara region in 2015 [33] (Table 3).

**Figure 7:**
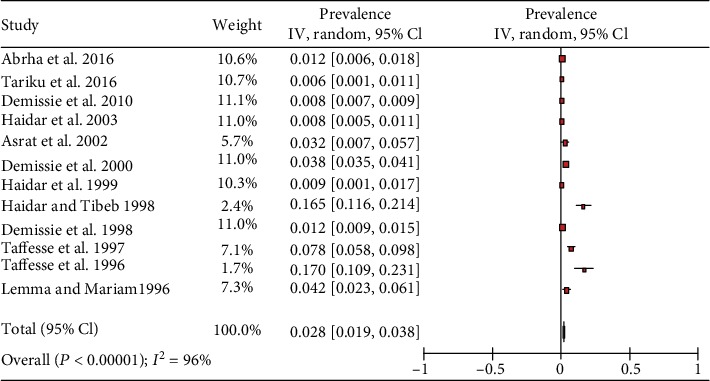
Forest plot of 13 studies on the prevalence of night blindness among preschool children in Ethiopia, 2019.

The overall prevalence of the meta-analysis of 13 studies, according to the Der Simonian-Laird random-effects model, revealed that the pooled prevalence of night blindness among preschool-age children in Ethiopia was 2.8% (95% CI: 1.9%-3.8%) (Figure 7). Subgroup analysis based on the survey year revealed that the prevalence of night blindness significantly decreased from 4.2% (95% CI: 2.8%-5.7%) in studies conducted from 1990 to 2004 to 0.8% (95% CI: 0.6%-1.0%) in studies conducted from 2005 to 2019. The prevalence of night blindness in moderate-risk studies was higher, 5.5% (95% CI: 3.3%-7.7%), than studies with a low risk of bias, 1.4% (95% CI: 0.8%-1.9%). In six studies, subgroup analyses by sex showed that the prevalence of night blindness among male and female preschool children was 4.6% (95% CI: 2.1%-7.1%) and 2.4% (95% CI: 0.9%-3.9%), respectively (Table 2).

The prevalence of Bitot's spot among preschool children in Ethiopia was higher from 1990 to 1993. A study conducted in 1991 in Hararge region documented 10.7% prevalence of Bitot's spot [29], while a study conducted in 1993 reported high prevalence of Bitot's spot (26.5%) among preschool children in Oromia region [24], whereas in 1997, it was 0.5% in a study done in the same district of Arsi zone [28]. Similarly, four studies [8, 25, 31, 32] conducted in Tigray region had shown a reduction of the prevalence of Bitot's spot ranging from 3.4% in 1993 [31] to 1.5% in 2014 [28]. Likewise, the prevalence of Bitot's spot in southwest Ethiopia was 2.1% in 1993 [30], while in 1995, it was 0.5% [34]. A study done in 1996 reported 7.6% and 1.5% Bitot's spot in Harari and Tigray regions, respectively [27]. The 2005 national survey reported 1.7% prevalence of Bitot's spot among preschool children in Ethiopia ranging from 0.7% in the Southern region to 3.2% in Amhara region [14]. Moreover, two recent studies [32, 33] conducted in Tigray and Amhara regions reported 1.5% and 2.9% of Bitot's spot among preschool children, respectively (Table 3).

The overall prevalence of the meta-analysis of 13 studies, according to the Der Simonian-Laird random-effects model, revealed that the pooled prevalence of Bitot's spot, among preschool-age children in Ethiopia, was 2.1% (95% CI: 1.3%-2.8%) (Figure 2). Subgroup analysis by survey year revealed that the prevalence of Bitot's spot in studies conducted from 1990 to 2004 was 2.2% (95% CI: 1.3%-3.2%), and in studies conducted from 2005 to 2019, the prevalence was 1.8% (95% CI: 1.2%-2.3%). The prevalence of Bitot's spot in moderate-risk studies was higher, 3.3% (95% CI: 1.6%-4.9%), than studies with a low risk of bias, 1.7% (95% CI: 0.9%-2.6%), while in six studies, subgroup analyses by sex showed that the prevalence of Bitot's spot among male and female preschool-age children was 2.1% (95% CI: 0.9%-3.4%) and 0.9% (95% CI: 0.1%-1.6%), respectively (Table 2).

### 3.4. Prevalence of Subclinical Vitamin A Deficiency

A total of 8 studies were included in the review to assess the prevalence of subclinical vitamin A deficiency among preschoolers in Ethiopia. Based on the modified serum retinol criteria for vitamin A deficiency criterion, VAD deficiency is <0.70 *μ*mol/l (<20 *μ*g/dl) and below is 0.35 *μ*mol/l representing severe VAD [22]. Accordingly, in a study conducted in Arsi zone Oromia of Ethiopia, the prevalence of severe vitamin A deficiency serum (plasma) retinol concentration cutoff value < 0.35 *μ*mol was 31.9%% in 1993 [24]. In a study in the Southern region of Ethiopia, lower prevalence of severe vitamin A deficiency (4.6%) was reported in 1996 compared to other studies [26]. Higher prevalence of severe vitamin A deficiency (23.6%) was reported from Harari and Tigray regions of Ethiopia in 1996 [27]. In two studies of Tigray region, the prevalence of severe VAD ranged from 6.8% to 16.1% [8, 25] (Table 4).

In a study conducted in the Arsi zone of Ethiopia, the prevalence of vitamin A deficiency serum (plasma) retinol concentration cutoff value < 0.7 *μ*mol was very high (80.9%) in 1993 [24]. In the study of the Southern region of Ethiopia, the prevalence of vitamin A deficiency was 28% in 1996 [26]. While in a study conducted in Harari and Tigray regions in 1996, it was 62.5% [27]. Likewise, the high prevalence of VAD deficiency was reported in two studies conducted in the Tigray region of Ethiopia which ranged from 43.8% in 1996 to 63.3% in 1997 [8, 25], while the 2005 national survey found 37.7% prevalence of subclinical VAD in Ethiopia. The highest rates were reported in Afar (57.3%) and Oromiya (56.0%) regions, and lowest prevalence rates were recorded in the Southern (11.3%) and the Tigray (14.3%) regions [14]. The 2015 national survey reported a relatively lower prevalence of vitamin A deficiency in Ethiopia (13.9%). Among the regions, the prevalence of VAD was high in the Harari region (21.0%) and moderate prevalence rates were recorded in other regions ranging from 10.3% in Amhara to 17.4% in Afar region [15]. However, in a study done in Southern Ethiopia in 2016, a relatively high prevalence of subclinical VAD (33.6%) was reported [25] (Table 4).

The overall prevalence of the meta-analysis of 8 studies, according to the Der Simonian-Laird random-effects model, revealed that the pooled prevalence of vitamin A deficiency, among preschool-age children in Ethiopia, was 45.4% (95% CI: 28.9%-61.9%) (Figure 3). Subgroup analysis by survey year revealed that the prevalence of vitamin A deficiency decreased from 55.7 (95% CI: 39.8%-71.6%) in studies conducted from 1990 to 2004 to 28.3% (95% CI: 9.8%-46.7%) in studies conducted from 2005 to 2019. Subgroup analyses of five studies showed that the prevalence of vitamin A deficiency among male preschool-age children in Ethiopia was 35.1% (95% CI: 21.1%-49.0%) while among female preschool-age children, it was 27.8% (95% CI: 14.7%-40.9%). The prevalence of vitamin A deficiency in moderate-risk studies, 62.4% (95% CI: 44.2%-80.5%), was higher than that in studies with a low risk of bias, 35.2% (95% CI: 18.9%-51.5%) (Table 2).

## 4. Discussion

This systematic review and meta-analysis synthesized epidemiological evidence on vitamin A deficiency among preschoolers in Ethiopia from 1990 to 2019. Accordingly, studies conducted from 1990 to 1994 had shown that the prevalence of night blindness was very high, 16.5% in Hararge region [29] and 17% in Oromia region [24]. Based on WHO criteria [36] to define the prevalence of night blindness as a public health problem, Ethiopia had shown good progress in the reduction of night blindness from severe public health problem to moderate as indicated by most studies from 1995 to 2015 which ranged from 8.4% in the Harari region in 1996 [27] to low prevalence (0.6%) in Amhara region in 2015 [33]. Of the 13 studies included in the review, 5 studies showed a mild degree of public health problem (<1%), 5 studies showed moderate problems (1.0-4.9%), and 3 studies showed a severe degree of public health problem (≥5%). Micronutrient supplementations, immunization services, and nutrition education strategies had been implemented that might partially contribute to the reduction of micronutrient deficiencies in the country. Biannual distribution of VAS, deworming, and nutritional screening of under-five children was initiated in 2004 [37]. In addition to routine vitamin A delivery, measles immunization and VAS campaigns [38, 39] and promotion of exclusive breastfeeding and consumption of vitamin A-rich foods were also conducted to accelerate the control of measles and reduce morbidity associated with vitamin A deficiency [40].

Even though history of night blindness has been reported to be a valid index of vitamin A deficiency among preschool children, its usefulness is questionable because of its subjectivity and misconception of the symptoms especially in developing countries [36, 41]. Given the limitations, the prevalence of night blindness significantly decreased from moderate public problem 4.2% (95% CI: 2.8%-5.7%) in studies conducted from 1990 to 2004 to mild public health problem 0.8% (95% CI: 0.6%-1.0%) in studies done from 2005 to 2019. This might be attributed to better management of diseases, increased immunization coverage, and routine vitamin A supplementation with the expanded immunization program through health extension program [38, 40]. Systematic reviews also indicated that vitamin A supplementation reduced the prevalence of vision problems, including night blindness and xerophthalmia [6, 7].

The prevalence of Bitot's spot is more VAD specific, reliable, and easy to standardize. Regarding the prevalence of Bitot's spot, from 1990 to 1995, the prevalence was unacceptably high in studies conducted in Hararge region in 1991 (10.7%) [29] and in the Arsi zone in 1993 (26.5%) [24]. However, from 1995 to 2018, the prevalence of Bitot's spot had shown a reduction, ranging from 0.2% [26] to 7.6% [27]. Knowing the seriousness of the problem, a disease-targeted nutrition education and vitamin A supplementation interventions had been implemented since 1989 and mass vitamin A capsule distribution campaigns were carried out in all affected villages in 1993 [42]. The prevalence of Bitot's spot in studies conducted from 1990 to 2004 was 2.2% (95% CI: 1.3%-3.2%) and remained almost constant in studies conducted from 2005 to 2019, 1.8% (95% CI: 1.2%-2.3%). This indicated that the prevalence of Bitot's spot is still higher than the threshold criteria set by WHO [21]. Of the 13 studies included in the current systematic review, 10 studies reported a prevalence of Bitot's spot above 0.5%, which indicates VAD remains a public health problem among preschool children in Ethiopia. This might be due to the high prevalence of morbidity, poor consumption of fruits and vegetables, the monotonous cereal-legume diet, poorer access to and lower consumption of vitamin A-rich foods, and low vitamin A supplementation [13, 43].

Since the clinical manifestations occur after serious damage to internal tissue, subclinical assessment is mandatory. Based on modified WHO criteria, serum retinol concentration < 0.70 *μ*mol/l (<20 *μ*g/dl) is considered subclinical VAD and below 0.35 *μ*mol/l as a severe VAD [22]. Accordingly, the prevalence of severe VAD ranged from 4.6% [26] to 31.9% [20, 24]. Of the five studies that assessed severe VAD, four studies reported above 5% indicating a severe public health problem. The prevalence of vitamin A deficiency (<0.7 *μ*mol) ranged from moderate (13.9%) in 2015 [15] to very high (80.9%) in 1993 [24]. Although prevalence of subclinical VAD, 55.7% (95% CI: 39.8%-71.6%), in 1990 to 2004 decreased to 28.3% (95% CI: 9.8%-46.7%) in 2005 to 2019, it remains a severe public health problem. Subclinical VAD is considered a moderate and severe public health problem when 10% to 20% and more than 20% of children have serum retinol levels less than 0.7 *μ*mol/l, respectively [22]. According to these WHO recommendations, among eight studies, seven of them indicated a severe public health problem and one study showed the moderate public health problem of VAD among preschool children in Ethiopia. This might be attributed to poor consumption of fruits, vegetables, and vitamin A-rich foods and low vitamin A supplementation coverage [13, 44]. According to 2016 Ethiopian demographic and health survey report, only 25.7% of under-five years of age children consumed foods rich in vitamin A in 2011 [44] and only 45% of children age 6-59 months received vitamin A supplements [13].

Based on the 2005 national survey findings, subclinical VAD was a moderate public health problem in Tigray and Southern regions, while the remaining seven regions and the two city administrations (Addis Ababa and Dire Dewa) shows a severe public health problem [14]. The high levels of morbidity and the unavailability and low consumption of fruits and vegetables as well as low vitamin A supplementation (22.6%) might have contributed to the relatively high prevalence of vitamin A deficiency in most regions of the country. The low prevalence observed in the Tigray region may be attributable to the relatively high vitamin A supplementation coverage in Tigray (79.2%) at the time of the survey [14]. The better availability and higher consumption of fruits and vegetables could contribute to the low levels of vitamin A deficiency in the Southern region [13]. However, according to 2015 national survey results, subclinical VAD was a severe public health problem only in Harari region and it was a moderate public health problem in the remaining eight regions as well as in the two city administrations. The low prevalence observed in 2015 (13.9%) compared to 2005 (37.7%) may be attributable to the relatively high vitamin A supplementation coverage (63%) in 2015 [15] compared to 22.6% in 2005 [14].

In this review, although there is no significant difference, male preschool children were clinically and subclinically more deficient than the female counterparts. This might be related to differences in feeding practices. It might also suggest that the difference between the risk to male children and female children depends on the socioeconomic and cultural contexts of the communities studied [45, 46]. One possible partial explanation is that boys may be out more often further from home than girl children, thus increasing their susceptibility to diarrheal diseases and exposure to intestinal parasites, such as Giardia, Ascaris, and hookworm which in turn can affect vitamin A status by increasing loss of nutrients and reducing the dietary absorption of vitamin A [46–48].

The prevalence of clinical and subclinical vitamin A deficiency in both sexes is higher than the WHO cutoff point. Generally, this study showed that both clinical and subclinical VAD among preschool children in Ethiopia are still a public health problem. Several studies conducted in developing countries on the impacts of vitamin A supplementation had shown a significant reduction in the prevalence of Bitot's spot and improvement in the serum retinol concentration [31, 49–51]. Vitamin A supplementation is an effective intervention associated with a reduction of morbidity, mortality, and nutrition-related blindness in under-five children [6, 7, 52]. The WHO currently recommends vitamin A supplementation to 6- and 59-month-old children [11]. However, only less than half (45%) of children age 6-59 months received vitamin A supplements in 2016 [13]. Hence, VAS program needs to be strengthened further with a nutrition education strategy to reduce clinical VAD among preschool children. It is also critical to address the direct and underlying causes of VAD through effective interventions, including dietary diversity, breastfeeding, fortification, and hygiene.

In the meta-analysis, there are various sources of biases including inaccurate selection of study participants, data collection, analysis, and selective reporting of the study results that could affect the findings [53]. The subgroup analysis according to the risk of bias showed that the studies with a moderate risk of bias reflected a higher prevalence of the conditions studied compared with studies with a low risk of bias. That is, poor-quality studies could overestimate the prevalence of VAD. Methodological deficiencies of the studies could contribute to this higher reported prevalence. The observed sampling frame deficiency and lack of representation of the target population in the medium-risk researches could affect results of studies. For instance, in study conducted in Arsi zone [30], subclinical analysis was measured among all children with xerophthalmia and only 5% of children without xerophthalmia. This may have overestimated the subclinical VAD prevalence. Further, the sample is small in some of the studies due to improper sample size calculation and assumptions taken during sample size calculation that could contribute to the variations in prevalence. In addition, circulating serum retinol is reduced in the presence of inflammation and prevalence of VAD can be overestimated [54]. Therefore, in studies not adjusted for inflammation, VAD could be overestimated as a result of the presence of inflammation.

## 5. Strengths and Limitations

Inclusion of the studies that measured both clinical and subclinical vitamin A status and extensive searches using different searching strategies (manual and electronic) were the strengths of this review. Data extraction was also conducted using a predetermined tool and was extracted by two authors independently to minimize bias. Quality assessment was also done by two independent authors. There were also some potential limitations to this study. High heterogeneity was recorded among the included studies. The source of high heterogeneity could be because studies were conducted in different regions of Ethiopia. Subgroup analysis was not performed according to the regions since we could not find enough representative studies for each region. Since some of the reviewed studies did not report prevalence by variables like sex, resident, vitamin A supplementation status, and comorbidity infections, it limits us to do intensive subgroup analysis. High heterogeneity was addressed by using the random-effects model to compute pool prevalence. The random-effects model considers any heterogeneity embedded in the meta-analysis. However, many unexamined individual-level confounders, like children's health conditions (comorbid infections) and vitamin supplementary status, may also contribute to the heterogeneity of VAD prevalence among studies. Generally, the overall very low quality of evidence should be considered when interpreting the findings.

## 6. Conclusions

Although the prevalence of night blindness and Bitot's spot had shown a reduced pattern in the period from 1990 to 2019, still the magnitude remains a moderate public health problem while the subclinical vitamin A deficiency (serum value < 0.70 *μ*mol) is a severe public health problem in Ethiopia based on the available evidence. Thus, it is recommended to continue the ongoing intervention program and strengthen it through the newly initiated health extension program to further lower the magnitude of the problem. In addition to vitamin A supplementation, sustainable interventions such as dietary diversification, food fortification, exclusive breast feeding promotion, and nutrition education should be integrated and implemented effectively for sustainable prevention of VAD.

## Figures and Tables

**Table 1:** Characteristics of 15 studies included in a systematic review and meta-analysis, 1990-2019.

Author	Survey year	Place of the study	Sample size	Sampling procedure	Age in months	Assessment methods
Abebe [35]	2016	Offa district, Southern Ethiopia	110	Systematic random sampling	36-60	Subclinical
EPHI 2016	2015	Ethiopia	1,148	Multistage cluster sampling	6-59	Subclinical
Tariku et al. [33]	2015	Dembia district, Amhara region	681	Multistage random sampling	24-59	Clinical
Abrha et al. [32]	2014	Asgede-Tsimbla, Tigray region	1,230	Systematic random sampling	24-59	Clinical
Demissie et al. [14]	2005	Ethiopia	23,148	Multistage cluster sampling	6-71	Subclinical & clinical
Haider et al. 2003	1997	Two districts in Tigray region	4,770	Multistage sampling	6-72	Subclinical & clinical
Asrat et al. [28]	1997	Dodotana Sire district, Arsi zone	188	Multistage sampling	6-72	Subclinical & clinical
Demissie et al. [27]	1996	Tigray and Harary regions	15,087	Stratified multistage sampling	6-72	Subclinical & clinical
Haidar et al. [25]	1996	Alaje & Samre, Tigray region	5,253	Stratified multistage sampling	6-72	Subclinical & clinical
Demissie et al. [26]	1996	Southern Ethiopia	4,123	Systematic sampling	6-71	Subclinical & clinical
Getaneh et al. [34]	1995	Jimma town, Southwest Ethiopia	628	Simple random sampling	6-59	Clinical
Taffesse et al. [31]	1993	Agebe Woreda, Tigray region	678	Cluster random sampling	6-72	Clinical
Lemma and Mariam [30]	1993	Agaro town, Southwest Ethiopia	434	Random sampling	6-72	Clinical
Tafesse et al. [24]	1993	Arsi zone, Central Ethiopia	147	Random sampling	6-72	Subclinical & clinical
Haidar and Tibeb 1998	1991	Hararge region, Ethiopia	230	Systematic sampling	6-72	Clinical

**Table 2:** Subgroup analysis of the prevalence of clinical and subclinical vitamin A deficiency by risk of bias, sex, and survey year using the *I*^2^ test for heterogeneity, 2019.

Subgroups	Night blinding prevalence% (95% CI)	*I* ^2^ (%)	Bitot's spot prevalence% (95% CI)	*I* ^2^ (%)	Subclinical VAD prevalence% (95% CI)	*I* ^2^ (%)
Survey year						
1990-2004	4.2 (2.8-5.7)	98	2.2 (1.3-3.2)	98	55.7 (39.8-71.6)	99
2005-2015	0.8 (0.6-1.0)	11	1.8 (1.2-2.3)	48	28.3 (9.8-46.7)	99
Sex						
Male	4.6 (2.1-7.1)	91	2.1 (0.9-3.4)	97	35.1 (21.1-49.0)	97
Female	2.4 (0.9-3.9)	85	0.9 (0.1-1.6)	95	27.8 (14.7-40.9)	96
Risk of bias						
Moderate risk	5.5 (3.3-7.7)	98	3.3 (1.6-4.9)	99	62.4 (44.2-80.5)	97
Low risk	1.4 (0.8-1.9)	91	1.7 (0.9-2.6)	98	35.2 (18.9-51.5)	99

**Table 3:** Prevalence of xerophthalmia among preschool age children in Ethiopia, 1990 to 2019.

Author (ref)	Survey year	Sample size	Place of the survey	Xerophthalmia prevalence	
				Night blindness	Bitot's spot
Tariku et al. [33]	2015	681	Dembia district, Amhara region	0.6%	2.9%
Abrha et al. [32]	2014	1,230	Asgede-Tsimbla district, Tigray	1.2%	1.5%
Demissie et al. [14]	2005	23,148	Ethiopia	0.8%	1.7%
Haidar et al. [8]	1997	4,770	Two districts of Tigray region	0.8%	1.5%
Asrat et al. [28]	1997	188	Dodotana Sire district, Oromia	3.2%	0.5%
Demissie et al. [27]	1996	15,087	Harari and Tigray regions	3.8%	3.5%
Haidar et al. [25]	1996	5,253	Alaje & Samre districts, Tigray	0.9%	1.5%
Demissie et al. [26]	1996	4,123	In Southern region, Ethiopia	1.2%	0.2%
Getaneh et al. [34]	1995	628	Jimma town, Southwest Ethiopia	0%	0.5%
Taffesse et al. [31]	1993	678	Agebe district, Tigray region	7.8%	3.4%
Lemma & Mariam [30]	1993	434	Agaro town, Southwest Ethiopia	4.2%	2.1%
Tafesse et al. [24]	1993	147	Dodota district, Arsi zone, Oromia	17%	26.5%
Haider & Tibeb [29]	1991	230	Hararge region, Ethiopia	16.5%	10.7%

**Table 4:** Prevalence of subclinical vitamin A deficiency among preschool age children in Ethiopia, 2019.

Authors	Survey year	Sample size	Place of the survey	Prevalence of vitamin A deficiency	
				VAD (<0.7 *μ*mol/l)	SVAD (<0.35 *μ*mol/l)
Abebe [35]	2016	110	Offa district, Southern Ethiopia	33.6%	Not reported
EPHI [15]	2015	1148	Ethiopia	13.9%	Not reported
Demissie et al. [14]	2005	996	Ethiopia	37.7%	Not reported
Haidar et al. [8]	1997	281	In two districts of Tigray region	43.8%	6.8%
Demissie et al. [27]	1996	643	Harari and Tigray regions	62.5%	23.6%
Haidar et al. [25]	1996	248	Alaje & Samre districts, Tigray	63.3%	16.1%
Demissie et al. [26]	1996	197	Kambatta zone, Southern region	28.0%	4.6%
Tafesse et al. [24]	1993	147	Dodota district, Oromia region	80.9%	31.9%

Key: VAD = vitamin A deficiency; SVAD = severe vitamin A deficiency.

## Supplementary Materials

Supplementary Materials (1) Figure S1: map of Ethiopia depicting different regions. (2) Moher D, Liberati A, Tetzlaff J, Altman DG, the PRISMA Group (2009). Preferred Reporting Items for Systematic Reviews and Meta-Analyses: the PRISMA Statement. PLoS Med 6(7): e1000097. doi:10.1371/journal.pmed1000097. (3) Table: sample search strategy from PubMed database. Table: sample search strategy from Cochrane Library. (4) Table S1: risk of bias assessment of 15 included studies using the Hoy 2012 tool with ten criteria. (5) Table S2: GRADE quality of evidence profile for prevalence of clinical and subclinical vitamin A deficiency among preschool children in Ethiopia.
