# Supplementary material for: Prevalence of Vitamin A Deficiency among Preschool Children in Ethiopia: A Systematic Review and Meta-Analysis
Source: Biomed Res Int. 2020 Feb 27;2020:8032894. doi: 10.1155/2020/8032894 (PMC7073500; doi:10.1155/2020/8032894)
Supplement: Supplementary Materials — (1) Figure S1: map of Ethiopia depicting different regions. (2) Moher D, Liberati A, Tetzlaff J, Altman DG, the PRISMA Group (2009). Preferred Reporting Items for Systematic Reviews and Meta-Analyses: the PRISMA Statement. PLoS Med 6(7): e1000097. doi:10.1371/journal.pmed1000097. (3) Table: sample search strategy from PubMed database. Table: sample search strategy from Cochrane Library. (4) Table S1: risk of bias assessment of 15 included studies using the Hoy 2012 tool with ten criteria. (5) Table S2: GRADE quality of evidence profile for prevalence of clinical and subclinical vitamin A deficiency among preschool children in Ethiopia. [file 8032894.f1.zip › MAP of ethiopia.docx]

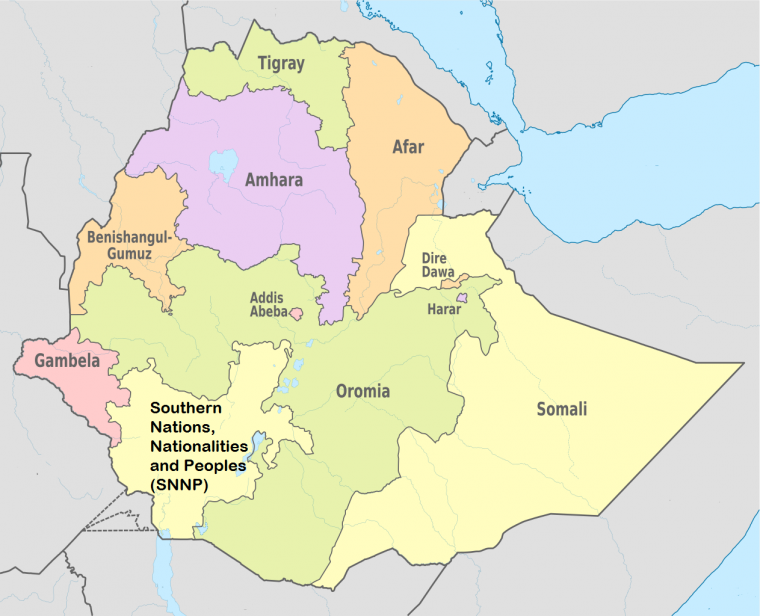


Fig S1:Map of Ethiopia depicting different regions

Source: <http://www.ethiovisit.com/ethiopia/ethiopia.html>
