# Supplementary material for: Prevalence of Vitamin A Deficiency among Preschool Children in Ethiopia: A Systematic Review and Meta-Analysis
Source: Biomed Res Int. 2020 Feb 27;2020:8032894. doi: 10.1155/2020/8032894 (PMC7073500; doi:10.1155/2020/8032894)
Supplement: Supplementary Materials — (1) Figure S1: map of Ethiopia depicting different regions. (2) Moher D, Liberati A, Tetzlaff J, Altman DG, the PRISMA Group (2009). Preferred Reporting Items for Systematic Reviews and Meta-Analyses: the PRISMA Statement. PLoS Med 6(7): e1000097. doi:10.1371/journal.pmed1000097. (3) Table: sample search strategy from PubMed database. Table: sample search strategy from Cochrane Library. (4) Table S1: risk of bias assessment of 15 included studies using the Hoy 2012 tool with ten criteria. (5) Table S2: GRADE quality of evidence profile for prevalence of clinical and subclinical vitamin A deficiency among preschool children in Ethiopia. [file 8032894.f1.zip › Supplemetary file 2 Quality of evidence.docx]

Table S2: GRADE quality of evidence profile for prevalence of clinical and subclinical Vitamin A deficiency among preschool children in Ethiopia

| **Quality assessment** | | | | | | | |
| --- | --- | --- | --- | --- | --- | --- | --- |
| **Number of  studies** | **Design** | **Risk of bias** | **Inconsistency** | **Indirectness** | **Imprecision** | **Publication bias** | **Overall quality of evidence** |
| **Prevalence of Night blindness among preschool children in Ethiopia** | | | | | | | |
| 12 | Cross-sectional | serious^1^ | serious^2^ | serious ^3^  indirectness | no serious imprecision | undetected | **VERY LOW**^1,2,3^ due to risk of bias, inconsistency and indirectness |
| **Prevalence of Bitot’s spot among preschool children in Ethiopia** | | | | | | | |
| 13 | Cross-sectional | serious^1^ | Serious^4^ | no serious indirectness | no serious imprecision | undetected | **VERY LOW**^1,4^ due to risk of bias and inconsistency |
| **Prevalence of subclinical Vitamin A deficiency** **among preschool children in Ethiopia** | | | | | | | |
| 8 | Cross-sectional | no serious risk of bias^1^ | Serious^5^ | no serious indirectness | no serious imprecision | undetected | **VERY LOW** ^5^ due to inconsistency |

^1^ 53.8% were from moderate risk of bias.
^2^ chi-squared tests of heterogeneity had a low p-value (p <0.05) and I2 value was 96%

^3^ chi-squared tests of heterogeneity indirect outcome (mothers/care givers reported a child cannot see in dim light)

^4^ chi-squared tests of heterogeneity had a low p-value (p <0.05) and I2 value was 97%

^5^ chi-squared tests of heterogeneity had a low p-value (p <0.05) and I2 value was 99%

An evaluation of the degree of certainty of the evidence for each outcome studied was performed using the GRADE (Grading of Recommendations Assessment, Development and Evaluation) tool. In GRADE approach observational studies start from low quality evidence but downgraded to very low based on the five factors; risk of bias, inconsistency, indirectness, imprecision and publication bias. Evidence from observational studies can be upgraded provided no other limitations have been identified based on the five factors. Assessments were made for the five main domains (risk of bias, consistency, directness, precision and publication bias), as well as overall quality of evidence. We used study design as our starting point and downgraded by one step for each domain that was not met; based on the GRADE recommendation

**Risk of bias** is limitations in the study design and implementation may bias the estimates of an intervention effect. Most information is from studies at low risk of bias: No serious limitations, No downgrade. The proportion of information from studies at high risk of bias is sufficient to affect the interpretation of results: Serious limitations, downgraded one level.

**Inconsistency** refers to an unexplained heterogeneity of results. Criteria to determine inconsistency can be applied when results are from more than one study and include: statistical criteria including tests of heterogeneity (e.g. chi-squared or Chi^2^) have a low p-value (p <0.05), [I^2^ value](mk:@MSITStore:C:\PROGRA~2\GRADEP~1\files\GRADEP~1.CHM::/Glossary%20of%20terms%20and%20concepts.htm#i2) is large (> 25%). Each outcome was downgraded by one due to inconsistency (tests of heterogeneity p <0.05 and I^2^ is large).

**Indirectness**: the question being addressed by the authors of a systematic review is different from the available evidence regarding the population, intervention, comparator, or an outcome. The quality of the evidence may be downgraded when substitute measurements or surrogate endpoints are measured instead of patient-important outcomes. Evidence for Night Blindness was downgraded because of indirect outcome (mothers/care givers reported a child cannot see in dim light) was used to assess child night blindness

**Imprecision:** results are imprecise when studies include relatively few patients and few events and thus have wide confidence intervals around the estimate of the effect. For dichotomous outcomes you should consider downgrading the quality of evidence because of imprecision for either of the following two reasons: The total sample size is lower than 2000   and/or total number of events is less than 300 (a threshold rule-of-thumb value) (based on: Mueller et al. [Ann Intern Med. 2007;146:878-881](http://www.annals.org/cgi/content/abstract/146/12/878)). Since the sample size is above 2000 and total events are 300 for each outcome. No down grade for imprecision

**Publication bias** is a systematic under-estimate or an over-estimate of the underlying beneficial or harmful effect due to the selective publication of studies. Funnel plot analysis, Egger weighted regression and Begg rank correlation tests were used to detect publication bias and P-value<0.05 was considered as indicative of statistically significant publication bias. The Egger weighted regression statistics of studies conducted on the prevalence of Night blindness (P= 0.052), Bigot’s Spot (P= 0.079) and subclinical vitamin A deficiency (p=0.078) and Begg rank correlation statistics (p > 0.05) indicated no evidence of publication bias. There was no sign of publication bias and asymmetry in the funnel plot. No down grade for Publication bias

The quality of evidence reflects the extent to which we are confident that an estimate of the effect is correct.

| **Grade** | **Definition** |
| --- | --- |
| High | Further research is very unlikely to change our confidence in the estimate of effect. |
| Moderate | Further research is likely to have an important impact on our confidence in the estimate of effect and may change the estimate. |
| Low | Further research is very likely to have an important impact on our confidence in the estimate of effect and is likely to change the estimate. |
| Very low | Any estimate of effect is very uncertain |
